# Hes1 Potentiates T Cell Lymphomagenesis by Up-Regulating a Subset of Notch Target Genes

**DOI:** 10.1371/journal.pone.0006678

**Published:** 2009-08-18

**Authors:** Darryll D. Dudley, Hong-Cheng Wang, Xiao-Hong Sun

**Affiliations:** Immunobiology and Cancer Research Program, Oklahoma Medical Research Foundation, Oklahoma City, Oklahoma, United States of America; New York University School of Medicine, United States of America

## Abstract

**Background:**

Hairy/Enhancer of Split (Hes) proteins are targets of the Notch signaling pathway and make up a class of basic helix-loop-helix (bHLH) proteins that function to repress transcription. Data from Hes1 deficient mice suggested that Hes1, like Notch1, is necessary for the progression of early T cell progenitors. Constitutive activation of Notch is known to cause T cell leukemia or lymphoma but whether Hes1 has any oncogenic activity is not known.

**Methodology/Principal Findings:**

We generated mice carrying a Hes1 transgene under control of the proximal promote of the lck gene. Hes1 expression led to a reduction in numbers of total thymocytes, concomitant with the increased percentage and number of immature CD8^+^ (ISP) T cells and sustained CD25 expression in CD4^+^CD8^+^ double positive (DP) thymocytes. Hes1 transgenic mice develop thymic lymphomas at about 20 weeks of age with a low penetrance. However, expression of Hes1 significantly shortens the latency of T cell lymphoma developed in Id1 transgenic mice, where the function of bHLH E proteins is inhibited. Interestingly, Hes1 increased expression of a subset of Notch target genes in pre-malignant ISP and DP thymocytes, which include Notch1, Notch3 and c-myc, thus suggesting a possible mechanism for lymphomagenesis.

**Conclusions/Significance:**

We have demonstrated for the first time that Hes1 potentiates T cell lymphomagenesis, by up-regulating a subset of Notch target genes and by causing an accumulation of ISP thymocytes particularly vulnerable to oncogenic transformation.

## Introduction

T cell development requires the productive assembly and expression of antigen receptor genes. For αβ T cells, expression of a functional T cell receptor β (TCR β) chain in complex with pre Tα protein on CD4^−^CD8^−^ (DN) thymocytes promotes cellular expansion and differentiation to the corresponding CD4+CD8+ (DP) stage through a CD8 immature single positive (ISP) stage [1], [2]. Next, functional rearrangement and expression of TCRα chains lead to formation of surface αβ TCR, which allows for signals that induce further development to mature CD4 or CD8 single positive (SP) T cells [3]. To ensure the development of T cells able to recognize MHC/peptide complexes, thymocytes are programmed to undergo apoptosis if they do not receive pre-TCR or TCR-mediated survival signals. Precise regulation of the signals that control proliferation vs. apoptosis is therefore critical for ensuring the proper differentiation of thymocytes that may be particularly vulnerable to oncogenic transformation during this highly dynamic phase of T cell development.

Notch signaling pathways regulate lineage specification decisions during development of numerous tissues [4]. Activation of transmembrane Notch receptors is triggered by interaction with Notch ligands Jagged and Delta-like on adjacent cells that results in proteolytic cleavage of Notch and subsequent release of the intracellular domain (IC) [5]. Notch-IC is then transported into the nucleus and associates with RBP-Jκ/CBF-1, resulting in the activation of target genes including the Hes family of proteins [6]–[8]. Notch mediates the development of T cells from multipotent lymphoid progenitors derived from bone marrow (BM) precursors that enter the thymus via the bloodstream [9]–[13]. Moreover, expression of a constitutively active form of Notch1 in BM progenitors results in ectopic T cell development outside the thymus and T cell leukemia [14], [15]. Hes1 is a basic Helix-Loop-Helix (bHLH) protein and forms homodimers to repress transcription by binding to N boxes and recruiting the transcriptional co-repressor Groucho [16], [17]. Data from analyses of Hes1 deficient mice suggested that Hes1 is required for normal T cell development, particularly in the expansion of early T cell progenitors [18]. On the other hand, overexpression of Hes1 in BM-derived progenitors impairs both myeloid and B lymphocyte differentiation [19].

T cell acute lymphoblastic leukemia (T-ALL) in humans is frequently associated with mutations or chromosomal rearrangements that result in Notch activation [20], [21]. Ectopic expression of activated forms of Notch induces rapid T cell transformation [15]. In childhood T-ALL, Tal1/SCL is often found to be expressed and thought to inhibit the function of bHLH proteins such as E2A and HEB, collectively called E proteins [22], [23]. Interestingly, a large fraction of these T-ALL cases accumulate activating mutations in the Notch1 gene [24]. Likewise, gain of function mutations within Notch are also found in several animal models of T-ALL such as T cell lymphoma developed in E2A deficient mice [25], [26]. Thus, Notch appears to cooperate with loss of E protein function in T cell tumorigenesis. The precise mechanism by which Notch activation leads to transformation is still unclear, though several potential targets have been identified [7], [27], [28]. To determine whether constitutive expression of Hes1 might contribute to T cell transformation, we have generated transgenic mice that express Hes1 from the proximal Lck promoter. We find that constitutive expression of Hes1 in the thymus leads to T cell lymphomas with low efficiency. However, Hes1 promotes rapid tumorigenesis in a well-studied model of T-ALL in which expression of Id1 inhibits the function of E2A and HEB transcription factors [29], whose deficiency leads to T cell lymphoma [30]. Therefore, we demonstrate for the first time that Hes1 itself possesses oncogenic activity. We also show that ectopic expression of Hes1 in pre-malignant ISP and DP thymocytes increases a subset of genes known to be activated by Notch signaling, such as Notch1, Notch3, and c-myc, thereby providing a potential mechanism by which Hes1 potentiates tumorigenesis.

## Materials and Methods

### Mouse models

To generate Hes1 transgenic mice, sequence encoding an HPC4 epitope tag was attached onto the 5′-end of murine Hes1 cDNA to facilitate detection of transgenic protein [31]. The construct was then cloned into the vector containing the proximal promoter of the lck gene [32]. The DNA fragment containing the transgene was injected into FVB/N blastocysts and two lines (H5 and H12) were selected for continued breeding and analysis. Id1 transgenic mice, in which the Id1 gene is expressed from the same lck promoter, were previously described as Id1-28 by Kim et al. [29].

### Flow Cytometry and cell sorting

Single cell suspensions from lymphoid tissues were stained with fluorochrome-conjugated antibodies and analyzed on a BD LSRII flow cytometer using standard procedures. To delineate DN thymocyte populations, thymocyte suspensions were stained with lineage specific antibodies for CD8, TCRγ/δ, B220, Gr1 and CD3. Upon gating on lineage negative cells, DN populations were further assayed based on CD44 and CD25 staining. The CD8 immature single positive (ISP) and DP cell populations were isolated based on CD4, CD8 and TCRβ expression after gating out all cells staining with propidium iodide. The ISP population was defined as CD4^-^CD8^+^TCRβ^lo^ cells. Cell sorting was performed on a MoFlo (Dako Colorado, Inc., Fort Collins, CO) using thymocytes from mice of different genotypes around 3 weeks of age.

### Real time PCR analysis

Total RNA from sorted T cells was isolated using Trizol reagent as per manufacturer's protocol. Five µg of total RNA was then used to synthesize cDNA using M-MLV reverse transcriptase (Invitrogen, Carlsbad, CA). Quantitative PCR was performed using SYBR green 1 (Qiagen, Valencia, CA) on an Applied Biosystems 7500 Real Time PCR and software analysis system. The primers used to detect Notch1, Notch3, Deltex1 and c-myc were purchased from Qiagen. Other primers are total Hes1F, CCAGCCAGTGTCAACACGA; total Hes1R,  AATGCCGGGAGCTATCTTTCT; endogenous Hes1F, TCCTTGGTCCTGGAATAGTGCTA; endogenous Hes1R, ACTGAGCAGTTGAAGGTTTATTATGTCT; Nrarp F,  CTACACATCGCCGCTTTCG; and Nrarp R, CGCGTACTTGGCCTTGGT.

### Sequence analysis of the Notch1 allele

Genomic DNA was isolated from cells of individual tumors, of which tumor cells constitute at least 90%, and used for PCR-mediated amplification of the PEST domain of the Notch1 gene with the following primers: TACCAGGGCCTGCCCAACAC and GCCTCTGGAATGTGGGTGAT. The resulting PCR products were separately cloned into the pGEM-T easy vector (Promega, Madison, WI). At least 5 colonies were analyzed by DNA sequencing to identify mutations.

### Statistical analyses

Student's *t* tests were performed using the Prism 5 software. Kaplan-Meier curves were constructed and the age of median survival was determined using the same software. Statistical significance was estimated with the log-rank test.

## Results

### Generation and characterization of Hes1 transgenic mice

**Figure 1:**
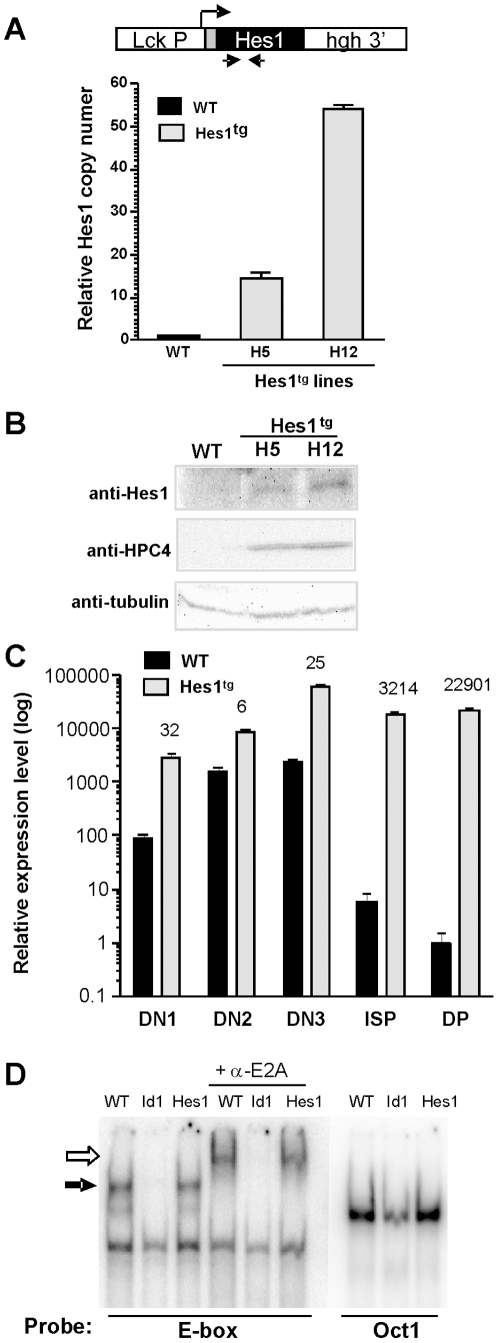
Generation and analysis of Hes1 transgenic mice. (A) Copy numbers of the Hes1 transgene determined using real-time PCR with primers as depicted by the arrows in the diagram of the transgenic construct. HPC4-tag was added to the N-terminus of Hes1. Numbers of Hes1 coding sequences in the H5 and H12 lines of Hes1 transgenic mice are shown as relatives to that of WT mice. (B) Hes1 protein expressed by total thymocytes from WT and the H5 and H12 lines of Hes1 transgenic mice were detected with antibodies against Hes1 or the HPC4 tag. Levels of tubulin were used as loading controls. (C) Comparison of total Hes1 mRNA levels encoded by the endogenous gene and transgene in thymocyte subsets sorted from WT and Hes1 (H12) transgenic mice. The level of Hes1 in each sample was normalized against that of β-actin. Expression levels relative to that in WT DP cells are shown in log scale. The fold of increase in Hes1 transgenic cells relative to their wild type counterparts is labeled on top of the bars. Data shown in (A) and (C) are means±SD of triplicates. (D) Electrophoretic mobility shift assay for E-box DNA-binding activity. Nuclear extracts were prepared from sorted DN thymocytes from the indicated mouse strains at the age of 3–4 weeks, and incubated at room temperature with ^32^P–labled probes and 1 µg of poly(dI:dC) as described [51]. For supershift assays, 1 µl of anti-E47 antibodies was added at the end of binding reaction and incubated for 5 minutes. DNA binding and super-shifted complexes are indicated by closed and open arrows. Oct-1 binding was used as a control.

We have generated transgenic mice in which Hes1 cDNA is expressed from the proximal promoter of the lck gene, which directs gene expression beginning at the DN stages of T cell development. Two independent lines, H5 and H12, were characterized and used in this study. Using quantitative PCR assays, we found that the H5 and H12 lines contained about 15 and 55 copies of the transgene, respectively (Fig. 1A). Correspondingly, we detected slightly higher levels of Hes1 protein level in the H12 line compared to the H5 line using antibodies against Hes1 or the HPC4 tag (Fig. 1B). The level of Hes1 in wild type thymocytes was undetectable with the anti-Hes1 antibodies commercially available. We next compared levels of total Hes1 transcripts in subsets of thymocytes (Fig. 1C). Although endogenous Hes1 was expressed at high levels in wild type DN thymocytes, its levels in ISP and DP cells were extremely low. In comparison, transgenic expression elevated total Hes1 levels by 6–32 folds in the DN compartments. However, the levels of Hes1 in ISP and DP thymocytes of the transgenic mice were 3,214 and 22,901 times higher than their wild type counterparts. These profound differences in Hes1 levels could have significant impact on the transgenic thymocytes.

Since Hes1 is a bHLH protein and suggested to serve as an inhibitor of E proteins [33], [34], we tested its ability to influence the DNA binding activities of E proteins using electrophoretic mobility shift assays and an E-box probe. Nuclear extracts were isolated from CD4^−^CD8^−^ DN thymocytes of 3 week-old wild type, Hes1 or Id1 transgenic mice (Fig. 1D). While the DNA binding activity in extracts isolated from Id1-expressing cells was almost completely abolished, the level of E-box binding complexes in Hes1 transgenic thymocytes was comparable to that detected in wild type cells. We also did not detect any new DNA binding complexes that might correspond to E protein and Hes1 heterodimers. Since the DNA binding activity was not reduced, E protein levels were unlikely diminished. Collectively, it appears that Hes1 did not significantly alter the function of E proteins in these transgenic mice.

### Ectopic Hes1 expression results in altered thymocyte development

**Figure 2:**
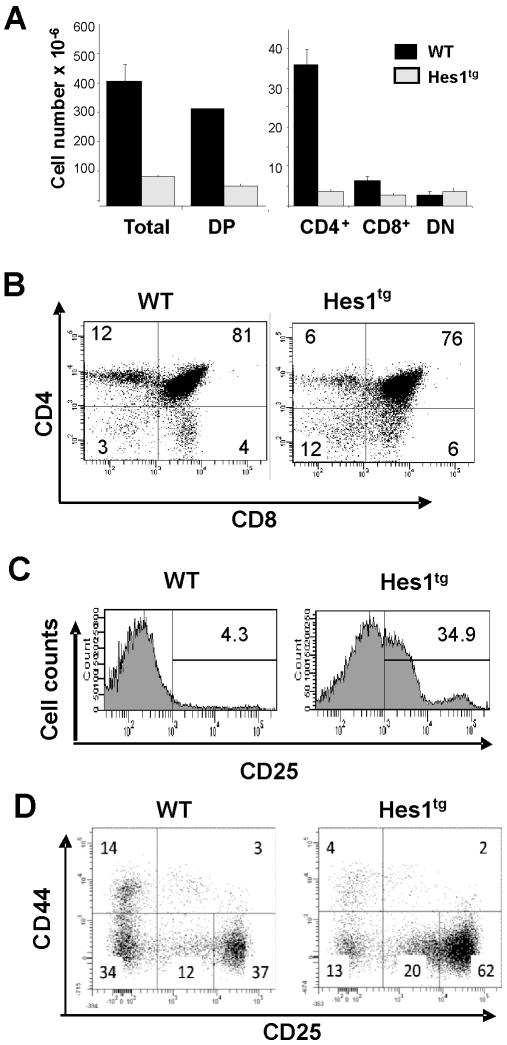
Analysis of thymocyte development in Hes1 transgenic mice. (A) Cell numbers of the different thymocyte populations are shown as an average of 4 mice at 3–5 weeks of age. (B) FACS analysis was performed on thymocytes isolated from individual wild type (WT) or Hes1 transgenic littermates and stained with indicated antibodies. (C) CD4^+^CD8^+^ DP cells shown in (B) were further analyzed for CD25 expression. (D) Thymocytes isolated from WT or Hes1 transgenic littermates were stained with antibodies against CD8, B220, TCRγ/δ, GR1 and CD3. The lineage negative thymocytes were further stained with anti-CD44 and anti-CD25. Percentages of cells in each gate are as labeled.

To investigate the effect of constitutive expression of Hes1 on thymocyte development, we analyzed thymocytes from 3 to 5-week old mice by flow cytometry and found an approximate 4-fold reduction in total thymocyte counts in Hes1 transgenic mice compared to littermate controls (Fig. 2A). This was accompanied by a decrease in the ratio of CD4 to CD8 SP thymocytes, (Fig. 2A and B). Although wild type CD4^+^CD8^+^ thymocytes no longer express CD25, the majority of Hes1 transgenic DP thymocytes express substantial levels of CD25 on their surface (Fig. 2C). The reduction in thymic cellularity was also accompanied by an increase in the percentage of CD4^−^CD8^−^ double negative cells (Fig. 2A and B). Furthermore, staining DN cells with antibodies against CD44 and CD25 revealed a consistently larger proportion of DN3 cells expressing intermediate levels of CD25 (20% versus 12%) in Hes1 transgenic mice (Fig. 2D). These cells could represent those transitioning from the CD44^−^CD25^+^ DN3 stage to the CD44^−^CD25^−^ DN4 stage following productive TCRβ rearrangement and functional pre-Tα expression. The percentages of DN4 cells were variable in individual Hes1 transgenic mice and were not significantly different from those of wild type mice.

**Figure 3:**
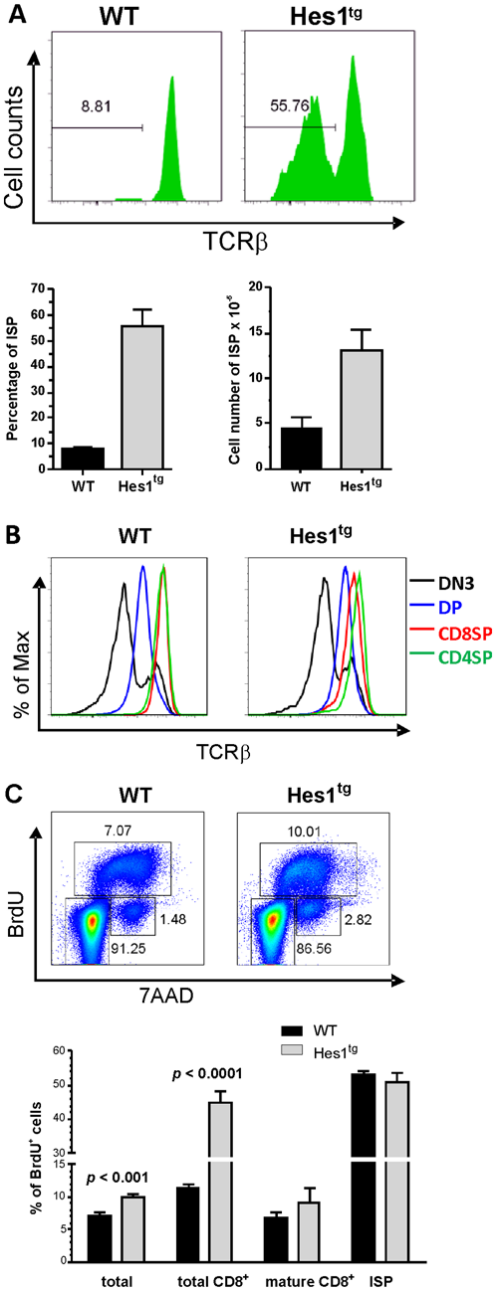
Accumulation of ISP in Hes1 transgenic mice. (A) FACS analysis was performed on thymocytes isolated from individual 3 to 5 weeks old WT or Hes1 transgenic littermates with antibodies against CD4, CD8 and TCRβ. The percentage of TCRβ^low/−^ cells in total CD8 single positive cells is shown in the histograms. Data shown are representatives of independent analyses of 7 WT and 11 Hes1 transgenic mice. Percentages of ISP in total CD8^+^ thymoyctes as well as absolute numbers of ISP thymocytes are shown in bar graphs as means±SE. (B) Thymocytes isolated from individual WT or Hes1 transgenic mice were stained for surface markers with antibodies against CD4, CD8, CD44, and CD25, which was followed by intracellular staining for TCRβ. The fluorescent intensities of color-coded populations in WT or Hes1 transgenic mice are overlaid in histogram. Representatives of independent analyses of multiple mice are shown. (C) Thymocytes isolated from individual WT or Hes1 transgenic littermates 1.5 hours after intraperitoneal injection with 1 mg BrdU. After staining with antibodies against CD4, CD8, CD44, and CD25, intracellular staining was performed using a labeling kit from BD Biosciences (San Jose, CA). Percentage of cells in each gate is indicated. Representatives of independent analyses of multiple mice are shown on the top. The percentage of BrdU labeling cells within indicated populations are shown on the bottom as means±SD.

One of the striking phenotypes of Hes1 transgenic mice was the accumulation of cells at the CD8 immature single positive (ISP) stage, which is an intermediate stage that occurs during the developmental transition from DN to DP stages [35], [36]. The ISP stage is characterized by low levels of TCRβ expression in comparison with that found on mature CD8^+^ thymocytes. We found a significant increase in the proportion of CD8^+^ thymocytes that expressed low levels of TCRβ in Hes1 transgenic mice compared to wild type littermates (Fig. 3A). Compared to wild type mice, the average percentages of the phenotypic ISP cells were increased by approximately 7 fold whereas the total numbers of these cells were 3 times higher in Hes1 transgenic mice (Fig. 3A). To rule out the possibility that these phenotypic ISP cells represent CD8^+^ cells without the rearrangement or expression of the TCRβ gene, we performed intracellular staining with antibodies against TCRβ. As shown in Fig. 3B, the peak level of intracellular TCRβ in CD8^+^ cells of Hes1 transgenic mice fell between those of DP and CD4^+^ cells. In contrast, the level in CD8^+^ cells of wild type mice was similar to those of CD4^+^ cells. This is consistent with the notion that a significant fraction of CD8^+^ cells expressed lower levels of TCRβ. However, if Hes1 transgenic CD8^+^ cells failed to express TCRβ, its staining intensity would have been similar to that of the major peak seen in DN3 cells (Fig. 3B). Therefore, we concluded that Hes1 transgenic mice have an accumulation of ISP cells.

Consistent with this finding, BrdU incorporation assay detected an increase in the percentage of BrdU positive cells in total thymocytes of Hes1 transgenic mice (Fig. 3C). Co-staining for CD4, CD8 and TCRβ with anti-BrdU showed that the proportion of BrdU positive cells in the total CD8^+^ population was dramatically different between wild type and Hes1 transgenic cells. However, when analyzed separately for mature CD8^+^ and ISP subsets, the percentages of BrdU incorporating cells were similar, suggesting that the larger fraction of BrdU positive cells in the total CD8^+^ population was due to the increased representation of ISP. These results thus suggested that Hes1 expression did not alter the rate of BrdU incorporation, which was also found to be similar in other thymocyte subsets (data not shown).

Collectively, constitutive expression of Hes1 impairs development of thymocytes at several stages. However, no significant abnormalities were detected in peripheral T cells, particularly in regulatory T cells, despite the elevated expression of CD25 in thymocytes (Fig. 2).

### Hes1 transgenic mice develop CD25^+^ thymic lymphomas

**Figure 4:**
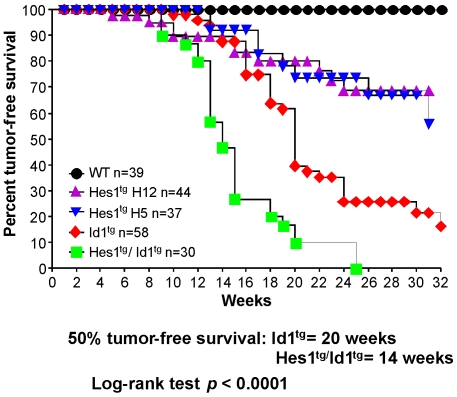
Incidence of thymic lymphomas in Hes1 and Id1 transgenic mice. Different strains of mice as indicated by symbols shown were monitored for onset of thymic lymphoma using outward signs such as respiratory distress. The presence of tumors was confirmed by autopsy. The cohort of Id1/Hes1 double transgenic mice included approximately equal representations of progenies of Id1 transgenic mice crossed with either the H5 or H12 line of Hes1 transgenic mice. Median time span of tumor free survival was calculated using the Prism software and shown below the graph. Statistical significance between the 50% tumor free survival in Id1 single and Id1/Hes1 double transgenic mice was determined using the Log-rank test. Numbers of animals in each cohort (n) are listed in the inset.

Ectopic expression of Notch proteins leads to the rapid development of thymic lymphomas in mice. Given that Notch induces the expression of Hes1 during thymopoiesis, we investigated whether Hes1 expression alone is sufficient for T cell transformation. T cell lymphomas were found in greater than 25% of either the H5 or H12 line of Hes1 transgenic mice with an average age of onset of 20 weeks (Fig. 4 and Table 1). Cell surface expression of T cell markers was found to vary between tumor samples, though the majority expressed both CD4 and CD8. The majority of lymphomas also expressed high levels of CD25, consistent with previous studies of Notch3-induced T cell lymphomas [37].

**Table 1:** Hes1 expression induces thymic lymphoma in mice.

Tumor #	Age at onset (weeks)	Tumor phenotype	Tissue involved
430	5	CD4^+^CD8^+^CD25^+^	Thy,Spl, LN
786	8	CD4^+^CD8^+^CD25^+^	Thy,Spl, LN
996	10	ND	Thy,Spl, LN
382	10.5	CD4^+^CD8^+^CD25^+^	Thy,Spl, LN
279	15	CD4^−^CD8^+^CD25^−^	Thy
662	15	CD4^+^CD8^+^CD25^+^	Thy
280	17	CD4^+^CD8^+^CD25^+^	Thy
798	22	CD4^+^CD8^+^CD25^+^	Thy
457	23	CD4^−^CD8^+^CD25^−^	Thy
254	24	CD4^+^CD8^+^CD25^+^	Thy
697	26	CD4^+^CD8^+^CD25^+^	Thy
741	31	CD4^−^CD8^+^CD25^−^	Thy,Spl, LN
633	47	ND	Thy

All mice that developed tumors had clear evidence of thymic involvement, though tumor cells were frequently detected in peripheral tissues including spleen and lymph nodes. Whenever spleen or lymph node involvement was detected, the surface marker phenotypes on leukemia cells were identical to that of cells isolated from the thymus of the same animal, suggesting that tumorigenesis originated in the thymus, which was followed by metastasis to peripheral lymphoid organs.

### Hes1 synergizes with Id1 in tumorigenesis

Models of T cell leukemia or lymphoma involving activated Notch display rapid onset leukemias that develop at about 6–8 weeks of age [14], [37]. In contrast, overexpression of Hes1 caused T cell lymphoma with a longer latency and lower penetrance (Fig. 4). This suggested that activation of Hes1 transcription by Notch signaling could not fully explain the potent oncogenic effects of Notch receptors. We thus explored possible cooperation between Hes1 and other factors in T cell lymphomagenesis. Tumorigenesis due to loss of E protein function is another well studied example of T-ALL [29], [30], [38], [39]. Previous studies have also shown that expression of Id1, a naturally occurring inhibitor of E proteins, leads to a high incidence of thymic lymphomas in mice [29]. Constitutive expression of Id1 from the lck proximal promoter leads to tumorigenesis with the age of 50% tumor free survival being 20 weeks (Fig. 4). When Hes1 transgenic mice were crossed with these mice, the resulting trans-heterozygotes expressing both Hes1 and Id1 developed thymic lymphomas at 100% frequency within 25 weeks and with a median onset of only 14 weeks (Fig. 4). The enhancement of tumorigenesis by Hes1 was statistically highly significant. Thus it appears that Hes1 and Id1 act synergistically to promote tumorigenesis.

### Hes1 up-regulates a subset of Notch target genes in pre-malignant thymocytes

**Figure 5:**
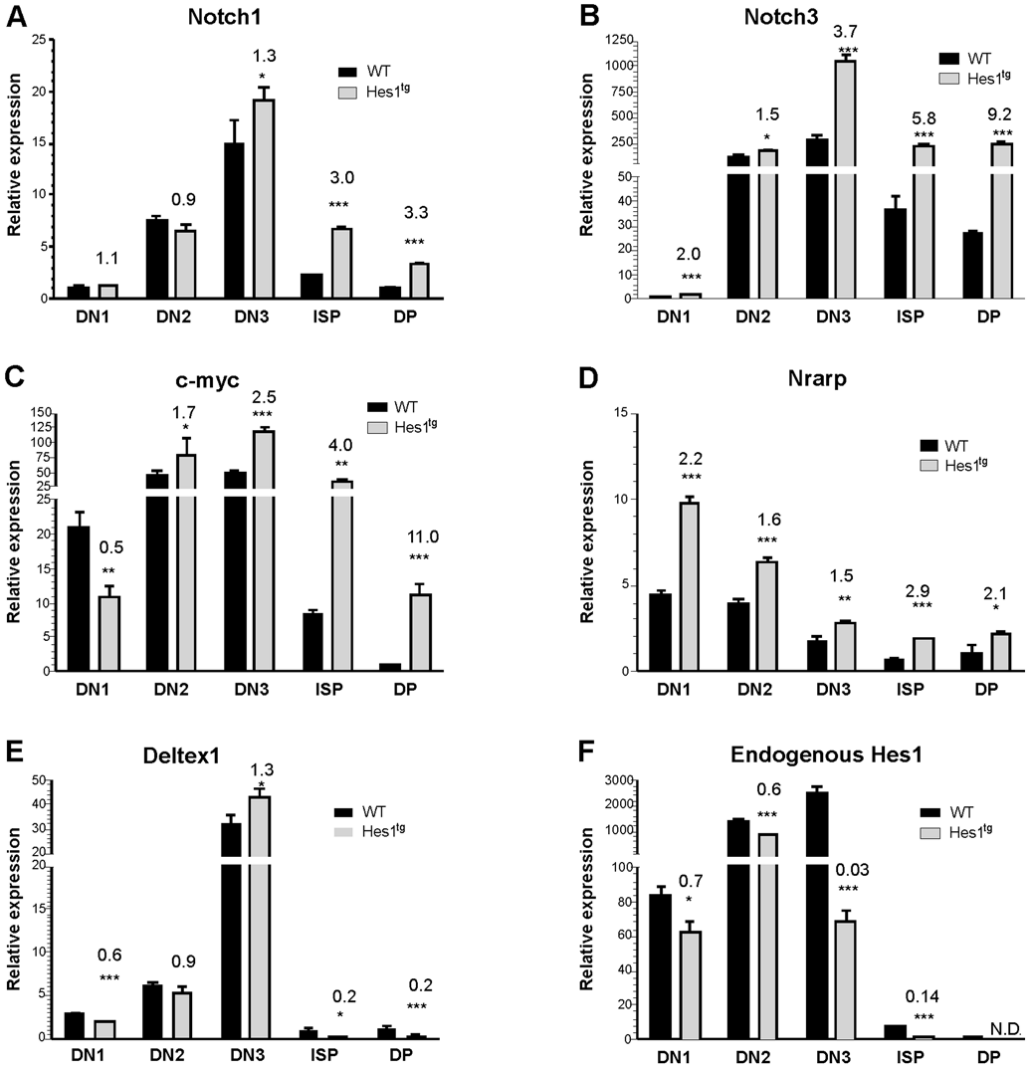
Ectopic expression of Hes1 elevates mRNA levels of a subset of Notch target genes. Real-time RT-PCR assays were performed using total RNA isolated from thymocyte subsets of WT and Hes1 (H12) transgenic mice as described for Fig. 1C. Relative levels of expression of each indicated transcript are presented in reference to that in WT DP cells. N.D. stands for not detectable. Data are means±SD of triplicates. The ratio of mRNA level in Hes1 transgenic versus WT cells is labeled on the top of the bar. Statistical significance was assessed with Student's *t*-test. *, P<0.05, **, P<0.01, ***, P<0.001.

To understand the mechanism whereby Hes1 promotes T cell lymphomagenesis, we tested whether overexpression of Hes1 might have similar effects as Notch signaling. We generated cDNA from RNA isolated from various thymocyte populations of 3 week-old wild type and Hes1 transgenic mice, which did not exhibit any sign of thymic lymphoma. We next performed quantitative RT-PCR assays of several Notch regulated genes. As shown in Fig. 5A and B, mRNA levels of both Notch1 and Notch3 in wild type thymocytes progressively increased in the DN compartments but dramatically decreased in ISP and DP subsets. However, expression of the Hes1 transgene sustained Notch1 and Notch3 expression in these subsets. Notch3 levels in ISP and DP cells of Hes1 transgenic mice were similar to the peak level of Notch3 found in wild type DN3 thymocytes. Hes1 expression had little or no influence on the already high levels of Notch1 and Notch3 in DN subsets.

Next, we examined the effect of Hes1 on expression of the c-myc gene, which is known to be a downstream target of Notch signaling [7], [8]. Like the expression patterns of Notch genes, c-myc levels were high in DN subsets of wild type mice and gradually decrease as the cells enter the ISP and DP stages (Fig. 5C). Hes1 again sustained c-myc expression in ISP cells at a level comparable to that seen in wild type DN3 cells. In DP thymocytes of Hes1 transgenic mice, the level of c-myc was 11 fold higher than their wild type counterparts. High levels of c-myc expression were also observed in ISP cells of Id1^tg^/Hes1^tg^ but not in Id1^tg^ mice (data not shown). Given the potent oncogenic effect of c-myc, up-regulation of c-myc by Hes1 could significantly contribute to the lymphomagenic effect of Hes1.

To address whether Hes1 expression uniformly enhances the transcription of all Notch targets, we interrogated additional target genes. Nrarp is considered a gene regulated by Notch and serves as a negative regulator of Notch signaling [40]. However, its expression pattern in wild type thymocytes did not follow the same trend as other Notch targets (Fig. 5D). Nevertheless, Hes1 increased Nrarp levels by less than 2.9 fold in various subsets. Surprisingly, two of the best known target genes of Notch signaling, Deltex1 and endogenous Hes1, were not stimulated by Hes1. Instead, transgenic expression of Hes1 resulted in reduction in most of the subsets. In particular, Deltex1 and endogenous Hes1 levels in the ISP faction were reduced by 5 and 7 fold, respectively. Therefore, it appears that ectopic expression of Hes1 selectively up-regulates a subset of but not all Notch target genes in pre-malignant thymocytes. Whether this is achieved by facilitating Notch-mediated transcription or through the action of independent mechanisms remains to be determined.

### Acquisition of stabilizing mutations of the Notch1 gene in Id1 and Hes1 expressing tumors

**Figure 6:**
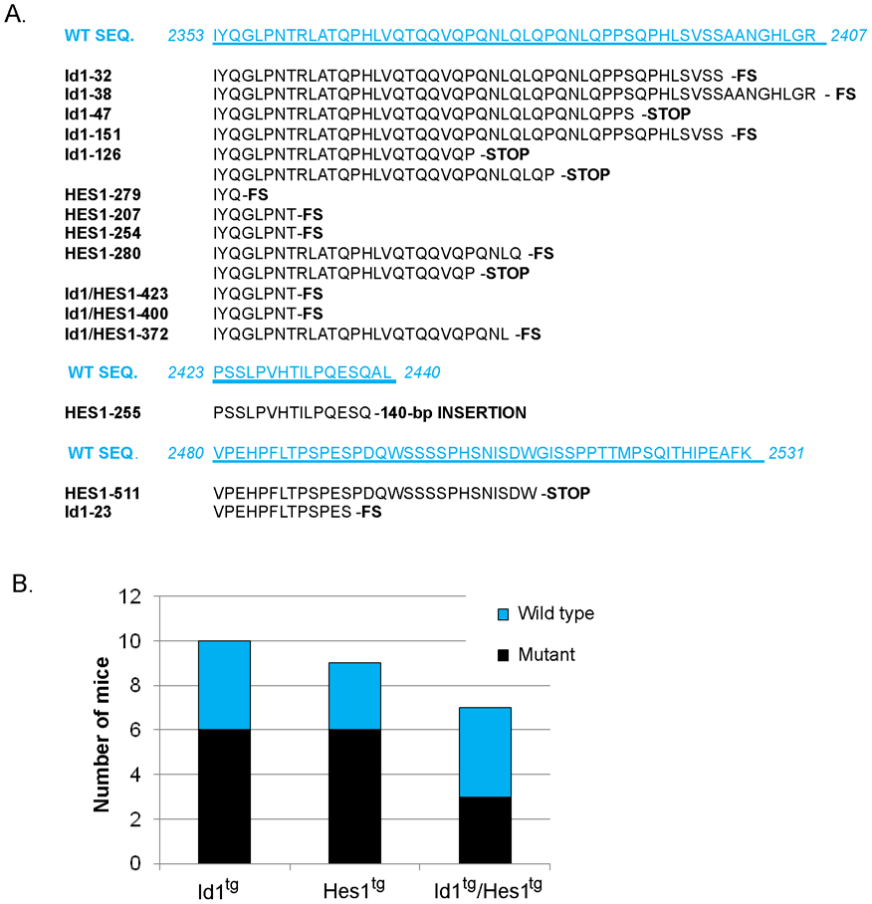
Mutations in the Notch1 gene from tumors developed in Id1 and Hes1 single or double transgenic mice. DNA samples isolated from individual tumors from indicated transgenic strains were used as templates to amplify the sequence encoding the PEST domain of Notch1. PCR products were cloned and sequenced as described in Materials and Methods. (A) Sequence of the mutations. Wild type Notch1 sequence in the relevant regions is shown in blue and amino acid numbers for each region are indicated. Mutant sequences from the tumors are aligned underneath and the type of the mutation is indicated at the end of each sequence (FS = frame-shift, Stop = nonsense mutation). (B) Frequency of Notch1 mutation. Numbers of tumors containing mutations and those with wild type sequence are depicted with black and blue bars, respectively.

Gain-of-function mutations in the Notch1 gene are frequently found in several models of T cell lymphoma as well as in human T cell leukemias, including those resulted from loss of E protein function [24]–[26]. We were therefore interested in learning if expression of one of the Notch targets, Hes1, could by-pass the need for mutations of Notch receptors. We surveyed 7–10 individual tumors developed in Id1 and Hes1 single or double transgenic mice for mutations in the PEST domain capable of de-stabilizing Notch1. PEST domain mutations represent the majority of mutations found in mouse T cell lymphomas and are thus good representatives. As shown in Fig. 6A, most of the alterations led to frame-shift or nonsense mutations in the gene, which is likely to abolish the function of the domain and cause stabilization of the mutant proteins. It is worth noting that there appear certain hotspots in PEST domain prone to mutations and most of the mutations occur at the N-terminus of the domain (Fig. 6A). Unexpectedly, similar percentages of tumors from Id1 and Hes1 transgenic mice (60% and 66%, respectively) accumulated mutations in the Notch1 gene (Fig. 6B). Three out of seven (43%) double transgenic tumors also contained mutations and this percentage, given the limited number of cases, is statistically insignificant compared to the 60–66% occurring in single transgenic tumors. Taken together, it appears that although Hes1 is able to turn on a subset of Notch targets, further enhancement of Notch signaling could provide advantages for tumor growth or survival. However, these PEST domain mutants alone are not sufficient to cause T cell leukemia [41].

### Analyses of gene expression in Hes1 transgenic tumors

**Figure 7:**
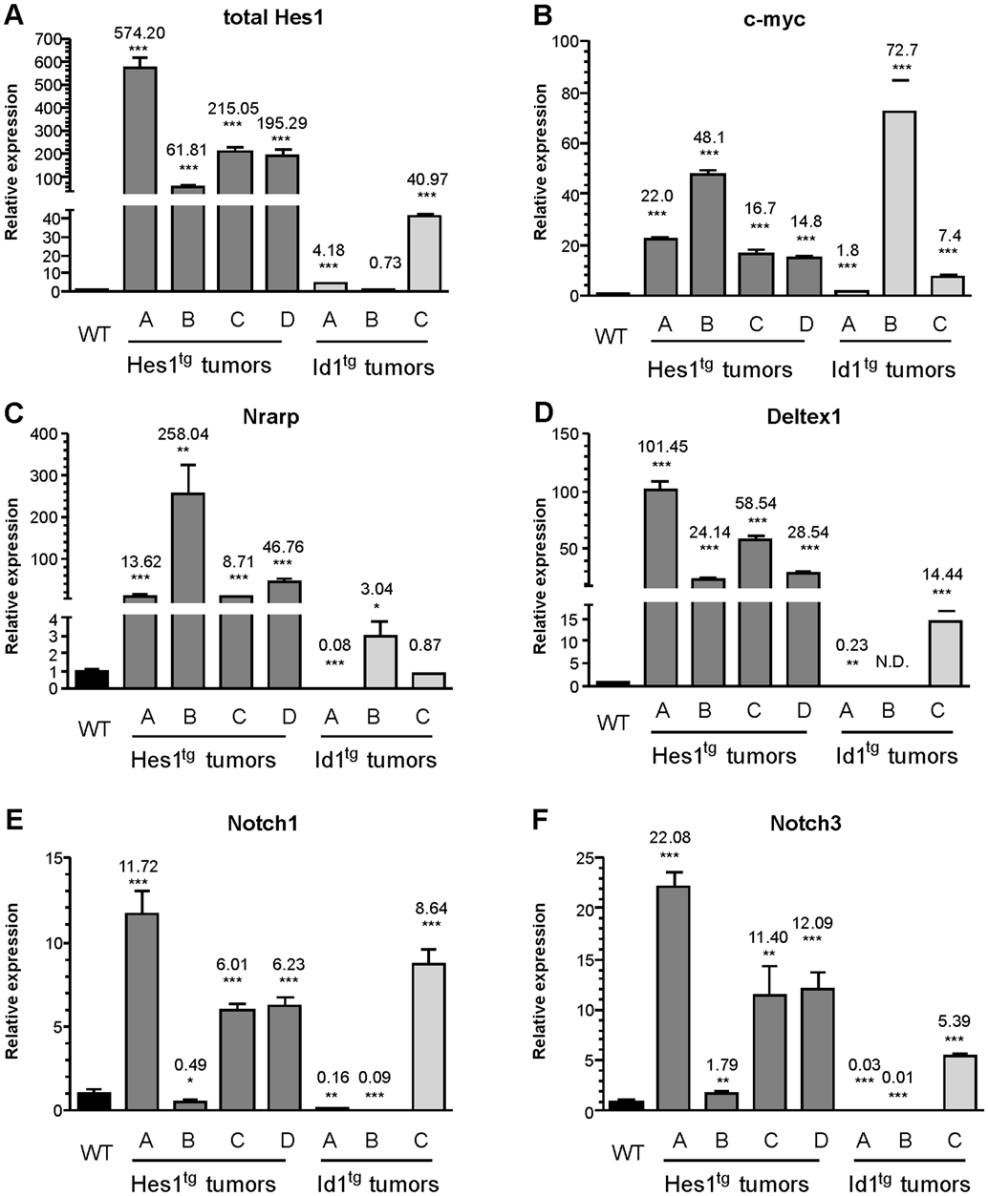
Analyses of gene expression in Hes1 transgenic T cell lymphomas. Total RNA was isolated from individual lymphoma samples of Hes1 or Id1 transgenic mice. Expression levels of indicated genes were determined in three independent experiments as described for Fig. 5 and compared to that in WT thymocytes.

To obtain further insights into the mechanism underlying tumor initiation or growth, we examined mRNA levels of Notch targets in the lymphomas. We sampled 4 Hes1 transgenic tumors along with 3 Id1 transgenic tumors as controls. Hes1 tumor A had a 140-bp insertion in the PEST domain that resulted in a frame-shift whereas the other three tumors did not have mutations in this region. Tumor A of Id1 transgenic mice did not carry a PEST domain mutation and Notch1 sequence in the other two tumors were not analyzed. All tumors from Hes1 transgenic mice produced high levels of Hes1 but Id1^tg^ tumors A and B had very low levels and tumor C expressed a moderate level of Hes1 (Fig. 7A). Consistent with uniformly high levels of Hes1, all Hes1^tg^ tumors expressed higher levels of c-myc (Fig. 7B). This agrees with our finding of elevated c-myc levels in pre-malignant ISP and DP thymocytes (Fig. 5). Although transgenic expression of Hes1 did not increase Deltex1 expression in pre-malignant thymocytes, elevated Deltex1 expression was seen in all Hes1^tg^ tumors and Id1^tg^ tumor C (Fig. 7D). Expression of Notch1 and Notch3 was elevated in Hes1^tg^ tumors A, C and D, as well as Id1^tg^ tumor C (Fig. 7E and F). Intriguingly, Hes1^tg^ tumor B had very low levels of Notch1 and Notch3, as well as lower levels of Hes1 and Deltex1 than other tumors. However, this tumor produced the highest levels of c-myc and Nrarp (Fig. 7B and C). Likewise, Id1 transgenic tumor B expressed a large amount of c-myc without up-regulating other Notch target genes except Nrarp. Collectively, elevation of different subsets of Notch target genes appears to occur in these T cell lymphomas of Hes1 transgenic mice. This probably represents the end result of natural selection for expression of a faction but not necessarily all of Notch targets, which in turn facilitate tumor cell growth and survival. No correlation between the expression patterns of these genes and the surface phenotypes of the tumors were detected in these analyses of limited numbers of samples.

## Discussion

Aberrant Notch signaling is well known to cause T cell leukemia or lymphoma with high efficiency. Hes1 is one of the first known target genes whose transcription is stimulated by Notch signaling [6]. Here, we show that ectopic expression of Hes1 predisposes mice to develop T cell lymphoma, which suggests that Hes1 up-regulation by Notch signaling is one of the components involved in Notch-mediated tumorigenesis. However, Hes1 transgenic mice develop lymphomas with a much longer latency and with a lower frequency compared to Notch1 or Notch3 expressing animals [37], [42]. This indicates that additional mechanisms besides Hes1 up-regulation play a role in Notch-induced tumorigenesis.

We have also demonstrated that Hes1 expression acts synergistically with Id1 to promote lymphoma development. Id1 is a naturally occurring inhibitor of bHLH E protein transcription factors such as E2A. E proteins are considered tumor suppressors because genetic ablation of the E2A gene results in T cell lymphoma [30]. Id1 expression also promotes T cell lymphomagenesis [29]. In addition, aberrant activation of Tal1, another bHLH inhibitor of E proteins, is the most common cause of human pediatric T cell acute lymphoblastic leukemia (T-ALL) [22], [43]. Transgenic expression of the p22 form of Tal1 also causes T cell lymphoma in mice [44]. When both Hes1 and Id1 are expressed in thymocytes, lymphomas develop rapidly in all animals examined (Fig. 4). The age of 50% tumor-free survival is 14 weeks. This efficiency is comparable to that seen in animals expressing Notch-IC [42]. Does this mean that T cell lymphoma resulting from constitutive Notch signaling involves both up-regulation of Hes1 and down-regulation of E protein function? This possibility is not entirely unreasonable because work from our laboratory has shown that Notch signaling accelerates ubiquitin-mediated degradation of E2A proteins in cellular environments with sufficient Erk activities [45], [46]. One can envision such a scenario in T cells immediately following pre-T cell receptor (pre-TCR) or TCR signaling. Notch signaling has also been shown to stimulate expression of Id genes such as Id1 or Id3 [47], [48]. Previous studies have revealed that loss of E protein function lowers the thresholds of TCR signaling [49]–[51]. Furthermore, Notch is known to facilitate T cell survival, which may contribute significantly to the manifestation of T cell lymphoma.

It has been shown that tumors developed from E2A deficient or Tal1 expressing T cells accumulate stabilizing mutations in the Notch1 gene [24], [25]. We found similar mutations in tumors expressing Id1, Hes1 or both (Fig. 6). This suggests that Hes1 expression cannot entirely substitute for enhanced Notch signaling during tumor progression. However, it is important to point out that ectopic expression of Hes1 did increase the frequency and shorten the latency of lymphomagenesis in Id1 transgenic mice. Hes1-mediated expression of a subset of Notch target genes occurred in pre-malignant thymocytes, which could constitute partial Notch signaling and lead to weak intrinsic oncogenic effects. Furthermore, Hes1 acts synergistically with Id1 to significantly enhance tumorigenesis in double transgenic mice. Although mutations in the Notch1 gene have been detected in various T cell leukemias or lymphomas triggered by loss of function of transcription factors or increases in signaling transduction [24], [25], [41], the exact role of these mutants is not well understood. For instance, the PEST domain mutants have very low activities in Notch-mediated transcription and T cell differentiation. They also fail to cause T cell leukemia when expressed from retroviral vectors [41]. In contrast, activation of a subset of Notch target genes occurs in pre-malignant thymocytes of Hes1 transgenic mice, which do not carry mutations in the PEST domain (data not shown). Therefore, the contribution of other oncogenic factors should not be dismissed simply because stabilizing Notch mutations are found in the tumors. These Notch mutants could merely cooperate with other oncogenic factors to promote the growth of existing tumor cells.

How does Hes1 potentiate tumor formation by itself and in cooperation with E protein inhibitors? Examination of T cell development in Hes1 transgenic mice revealed an accumulation of CD8 immature single positive cells. This stage coincides with the proliferative burst following pre-TCR expression, making cells uniquely susceptible to tumorigenesis. This is particularly relevant to the situations when E protein functions are compromised. For example, heterozygous Id1 transgenic mice, where T cell development is partially impaired, develop high incidence of T cell lymphoma [29]. However, homozygous Id1 transgenic mice, in which T cell development is blocked at the DN1 stage, do not develop T cell lymphoma (data not shown). On the other hand, conditional disruption of the E2A gene in DP thymocytes also did not result in thymic tumors [52], suggesting the window of opportunity for transformation occurs prior to this stage. It is also interesting that retrovirus-mediated expression of Hes1 in bone marrow cells does not promote tumorigenesis in transplant recipients [18], [19]. This may be explained by the differences in target cells and the timing of Hes1 expression.

At the molecular level, we have detected elevated levels of c-myc expression in pre-malignant thymocytes, particularly in the ISP and DP populations, where c-myc is normally expressed at low levels. It is well documented that c-myc exhibits potent transforming activity in T cell leukemia and lymphoma [53], [54]. The c-myc gene is a known target of the Notch signaling pathway [7], and with Hes1 being downstream from Notch receptor signaling, it is therefore plausible that Hes1 can cause c-myc up-regulation. Interestingly, c-myc, along with Notch1, Notch3 and CD25, is among a subset of Notch target genes to be stimulated by Hes1. In contrast, Deltex1 and endogenous Hes1 genes were not up-regulated in pre-malignant thymocytes by transgenic Hes1. Likewise, PTEN expression was not altered in pre-malignant thymocytes of Hes1 transgenic mice (data not shown). These findings argue against the idea of a uniform elevation of Notch signaling in pre-malignant Hes1 transgenic thymuses. Instead, they suggest that different Notch target genes are regulated differently, or at least, have different thresholds for Notch signals. This notion is further reaffirmed by data obtained from a limited analysis of gene expression in Hes1 transgenic tumor samples, showing expression of a combination of different Notch targets at varying levels in different tumors.

Despite the positive effects of Hes1 on gene expression, Hes1 is known to repress transcription by binding to N-box DNA sequences and recruiting the transcriptional co-repressor, Groucho [16], [17]. How Hes1 conversely brings about increases in gene expression needs to be understood in the future. Up-regulation of Notch targets by Hes1 constitutes a positive feedback for Notch signaling. Elucidation of the molecular mechanisms by which Notch signaling and its downstream effectors influence tumorigenesis will facilitate therapeutic intervention of T cell leukemia and lymphoma and also shed light on the oncogenic effect of Notch on other cell types.
